# The diagnostic efficiency of artificial intelligence based 2 hours Holter monitoring in premature ventricular and supraventricular contractions detection

**DOI:** 10.1002/clc.24266

**Published:** 2024-04-08

**Authors:** Qiong Huang, Yuansheng Fan, Jialin Wang, Zhiyang Xu, Linfeng Yang, Junhong Wang, Yiyang Zhan, Xiangqing Kong, Ningtian Zhou

**Affiliations:** ^1^ Department of Cardiology The First Affiliated Hospital of Nanjing Medical University Nanjing Jiangsu China; ^2^ Department of Cardiac Surgery The Second Affiliated Hospital of Nanjing Medical University Nanjing Jiangsu China; ^3^ Department of General Practice The First Affiliated Hospital of Nanjing Medical University Nanjing Jiangsu China; ^4^ Department of Geriatrics The First Affiliated Hospital of Nanjing Medical University Nanjing Jiangsu China

**Keywords:** arrythmia, artificial intelligence, diagnostic efficiency, Holter monitoring

## Abstract

**Background:**

Electrocardiography (ECG) and 24 hours Holter monitoring (24 h‐Holter) provided valuable information for premature ventricular and supraventricular contractions (PVC and PSVC). Currently, artificial intelligence (AI) based 2 hours single‐lead Holter (2 h‐Holter) monitoring may provide an improved strategy for PSVC/PVC diagnosis.

**Hypothesis:**

AI combined with single‐lead Holter monitoring improves PSVC/PVC detection.

**Methods:**

In total, 170 patients were enrolled between August 2022 and 2023. All patients wore both devices simultaneously; then, we compared diagnostic efficiency, including the sensitivity/specificity/positive predictive‐value (PPV) and negative predictive‐value (NPV) in detecting PSVC/PVC by 24 h‐Holter and 2 h‐Holter.

**Results:**

The PPV and NPV in patients underwent 2 h‐Holter were 76.00%/87.50% and 96.35%/98.55, respectively, and the sensitivity and specificity were 79.17%/91.30%, and 95.65%/97.84% in PSVC/PVC detection compared with 24 h‐Holter. The areas under the ROC curves (AUCs) for PSVC and PVC were 0.885 and 0.741, respectively (*p* < .0001).

**Conclusions:**

The potential advantages of the 2 h‐Holter were shortened wearing period, improved convenience, and excellent consistency of diagnosis.

## Graphical abstract

This study compared an artificial intelligence (AI) (convolutional neural network [CNN])‐based 2 hours single‐lead Holter and a traditional 24 hours Holter for premature supraventricular contraction (PSVC)/premature ventricular contraction (PVC) detection. The two methods showed good diagnostic consistency. In conclusion, the AI‐based 2 hours single‐lead Holter improved the PSVC/PVC detection efficiency, shortened the wearing period, and provided new insights for future arrhythmia diagnosis.

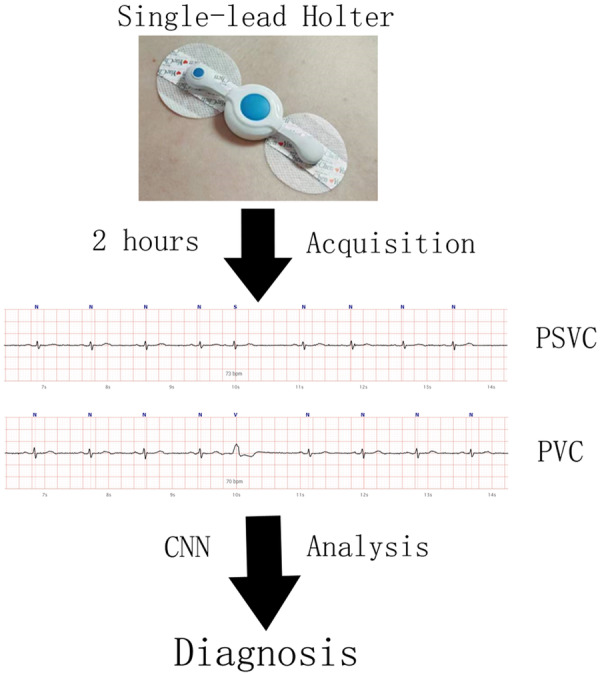

## 1 INTRODUCTION

Arrhythmias have serious implications for patients' health.^1^ Timely and accurate diagnosis of arrhythmias is crucial for their effective treatment and management.^2^ The use of electrocardiography (ECG) and 24 hours Holter monitoring (24 h‐Holter) has become increasingly important in the diagnosis of arrhythmias; however, both are limited to hospital.^3^, ^4^ In recent years, community arrhythmia screening programs have been widely performed in Chinese primary medical institutions, including ECG, ambulatory monitoring, and smartphone‐based technologies.^5^ The effectiveness of these methods in detecting arrhythmias varies, and considerations such as the cost, accessibility, and scalability should be considered when selecting a screening approach. Despite this, most medical institutions still choose ECG and Holter tests as the primary tools for arrhythmia screening. Although valuable information can be captured, this limitation has been obviously.^6^, ^7^

ECG provides valuable information about heart rhythm and structure. However, ECG has limitations in detecting arrhythmias that occur infrequently or unpredictably.^8^, ^9^ Because ECG is typically performed for a short duration, it cannot capture intermittent arrhythmias that may be critical for accurate diagnosis. As an arrhythmia diagnostic hallmark, Holter monitoring is a continuous ECG recording over a 24–48 hours period.^10^, ^11^ This allows for the detection of arrhythmias that occur intermittently. However, Holter monitoring has limitations in terms of patient compliance and discomfort owing to wearing the device for an extended period.^12^ To overcome the limitations of ECG and Holter monitoring, event monitors, implantable loop recorders, and other wearable applications appeared and spread in last decade.^13^, ^14^ In particular, the artificial intelligence (AI)‐based single‐lead Holter test exhibits outstanding comfortability and accessibility.^15^ Only one patch that adhered to the V2 location on the chest was easy to accept.

In previous studies, convolutional neural networks (CNN) have shown great potential as mature AI algorithms in ECG analysis. One common method involves preprocessing the ECG signals, such as filtering and noise reduction, before feeding them into the CNN.^16^ The CNN then learn to extract relevant features from the ECG signals, such as QRS complexes or ST‐segment abnormalities.^17^ These features can be used for various tasks such as arrhythmia detection, heart rate variability analysis, and myocardial infarction diagnosis.^18^

In this study, we focused on the diagnostic efficiency of 2 hours short‐term AI/CNN‐based Holter monitoring (2 h‐Holter) for premature ventricular and supraventricular contractions (PVC and PSVC), which are among the most common arrhythmias.^19^, ^20^ 2 h‐Holter analysis was used to predict PSVC and PVC in patients with unknown arrhythmia and was subsequently compared with 24 h‐Holter results to assess the sensitivity, specificity, positive‐predictive value (PPV), and negative predictive‐value (NPV) in detecting PSVC/PVC. Therefore, the present study was performed to address the aforementioned issues and assess a possible association between the diagnostic accuracy of 2 h‐Holter monitoring and 24 h‐Holter monitoring. The present study provides an improved strategy for AI diagnosis of PSVC/PVC.

## 2 METHODS

### 2.1 Study population

The Ethics Committee of the First Affiliated Hospital of Nanjing Medical University (Nanjing, China) approved this study, and all patients provided written informed consent (approval no. 2020‐SRFA‐301). All methods were performed in accordance with relevant guidelines and regulations, and informed consent was obtained from all participants and/or their legal guardians (s). In total, 170 patients were enrolled between August 2022 and August 2023 at The First Affiliated Hospital of Nanjing Medical University (Nanjing, China). None of these patients had a history of heart failure, fatal diseases, malignant arrhythmia, or an echocardiograph indicative of myocardiopathy. Before Holter monitoring, all patients underwent resting ECG and cardiac physical examination to exclude contraindications, such as chest malformation and local skin damage. All patients wore the traditional 24 h‐Holter and single‐lead AI 2 h‐Holter applications at the same time. The patients' medical information, including ECG/24 h‐Holter/2 h‐Holter results, were recorded and subsequently analyzed using the SWCAN AI‐ECG diagnostic platform (Nanjing, China) and verified by three independent cardiologists.

### 2.2 Diagnostic criteria

A PSVC/PVC frequency of ≥60/2 h‐Holter or 720/24 h‐Holter was defined as positive. A total of 24 patients were diagnosed with PSVC, 23 patients with PVC, and 115 patients were diagnosed negative by 24 h‐Holter.

### 2.3 12‐lead ECG

In the 12‐lead ECG, electrodes were placed at specific locations on the chest to obtain different views of the heart. Standard electrode placement included the right arm (RA), left arm (LA), right leg (RL), and left leg (LL) as limb leads. Additionally, six precordial leads (V1–V6) were placed on the chest in specific positions to capture electrical activity from different angles.

### 2.4 Twenty four hours‐Holter monitoring

Holter monitoring is a reference standard used to record the electrical activity of the heart over 24 hours. During the procedure, the patient wore a portable device called a Holter monitor, which was connected to electrodes placed on the chest. The monitor continuously records the electrical signals of the heart, allowing doctors to analyze any abnormalities or irregularities in heart rhythm. This procedure is often used to diagnose and monitor conditions, such as arrhythmias and heart palpitations.

### 2.5 Two hours single‐lead Holter monitoring

The single‐lead Holter is a wearable device (SWCAN‐SWK801, Nanjing) that records the electrical activity of the heart for over 2 hours. It consists of electrodes attached to the chest (CM2 location) and connected to a small recording device using Bluetooth 5.0. The patient wore the device throughout the monitoring period, allowing for continuous monitoring of the electrical signals of the heart. This can provide valuable information about heart rhythm and any abnormalities that may be present.

### 2.6 CNN algorithm‐based analysis system

According to our preprint article disclosed in the Research Square (https://doi.org/10.21203/rs.3.rs-2709337/v1). All 2 h‐Holter ECG data were stored and analyzed in real‐time using the CNN algorithm. CNN are a specialized type of neural network designed to process data with a known grid‐like topology. The network consists of eight residual blocks, each containing the following five layers:

1. A convolution layer
2. A batch normalization layer
3. A rectified linear unit (ReLU) layer
4. Another convolution layer
5. Another batch normalization layer

Both convolution layers (1 and 4) used a 3 × 3 kernel and stride of 1. After each convolution, batch normalization is applied to prevent parameter explosion and the vanishing gradient problem. Following the batch normalization layer, we added the ReLU activation function, which outputs zero for negative inputs and retains positive inputs. This nonlinearity allows the network to create complex nonlinear representations of the ECGs for automatic feature extraction. In addition to the above five layers, the residual blocks also include a residual connection that enables gradient propagation using a 1 × 1 convolutional layer between the input and output of the residual block. Between any two neighboring residual blocks, ReLU activation is used to filter the output of the previous block to the next block. After the last residual block, the data were fed into a global average pooling layer, followed by a fully connected layer, to generate the probability of each type of arrhythmia. The data set was split into training, validation, and test sets in a ratio of 8:1:1. There was no patient overlap between the sets.

### 2.7 Statistical analysis

SPSS (version 22.0; IBM Corp.) and GraphPad Prism 5 (GraphPad Software, Inc.) were used for the statistical analyses. Data are presented as percentages, frequencies, or mean ± SD. The Student's unpaired two‐tailed *t* test and *χ* ^2^ test were used to assess differences between groups. A two‐tailed *p* < .05 was considered statistically significant.

Receiver operating characteristic (ROC) curve analysis was performed to determine the specificity and sensitivity in the diagnosis of PSVC and PVC. Bland‐Altman analysis was used to compare the differences between 24 h‐Holter and 2 h‐Holter in the PSVC/PVC group. The following equations were used: PPV = (true positive)/(true positive + false positive) and NPV = (true negative)/(true negative + false negative).

## 3 RESULTS

### 3.1 Study population

Between August 2022 and August 2023, 170 patients were enrolled in the Cardiology Department of the First Affiliated Hospital of Nanjing Medical University (Nanjing, China). All the patients complained of inpatient or outpatient palpitations. ECG, 24 h‐Holter and 2 h‐Holter monitoring were used to diagnose arrhythmias. In the Negative Control Group (NC), PSVC group, PVC group and PSVC + PVC group, the mean age was 65.04 ± 12.23, 66.21 ± 15.43, 67.13 ± 16.83, 69.88 ± 11.00 years, respectively. Other baseline comparisons included body mass index, blood pressure (SBP and DBP), hypertension, diabetes mellitus, and coronary artery disease. None of the baseline characteristics were significantly different between the two groups (*p* ≥ .05). However, partial specific indicators, such as ejection fraction, free triiodothyronine, and thyroid‐stimulating hormone, were significantly different (*p* < .05). The demographic and clinicopathological characteristics of the patients are presented in Table 1.

**Table 1:** Clinical characteristics of the study subjects.

	Sex (males/females)	Age (years)		BMI (kg/m^2^)	EF (%)	SBP (mmHg)	DBP (mmHg)	HTN	DM	CAD	FT3 (pg/ml)	FT4 (pg/ml)	TSH (uIU/ml)
NC (115)	63/52	65.04 ± 12.23		24.39 ± 3.699	61.22 ± 7.555	127.3 ± 18.76	75.49 ± 11.72	36	23	23	4.497 ± 1.134	16.96 ± 3.498	2.733 ± 2.351
PSVC (24)	10/14	66.21 ± 15.43		24.26 ± 4.071	63.68 ± 2.943	127.0 ± 16.32	72.92 ± 11.57	9	6	6	4.216 ± 0.7235	16.47 ± 2.366	2.061 ± 1.266
PVC (23)	15/8	67.13 ± 16.83		23.91 ± 2.434	51.34 ± 14.08	122.2 ± 18.34	72.57 ± 9.741	7	5	6	3.685 ± 0.6664	16.71 ± 2.565	4.091 ± 3.922
PSVC + PVC (8)	5/3	69.88 ± 11.00		23.31 ± 2.841	55.80 ± 12.82	126.4 ± 22.48	67.38 ± 11.80	4	3	1	3.935 ± 0.3306	17.82 ± 2.033	1.391 ± 0.3784
**P**	0.4123	0.7168		0.8184	<0.0001	0.6973	0.1740	0.6858	0.6774	0.8016	0.0036	0.7508	0.0128

Abbreviations: BMI, body mass index; CAD, coronary artery disease; DBP, diastolic blood pressure; DM, diabetes mellitus; EF, eject fraction; FT3, free triiodothyronine; FT4, free thyroxine; HTN, hypertension; SBP, systolic blood pressure; TSH, thyroid stimulating hormone.

### 3.2 Diagnostic efficiency

**Figure 1:**
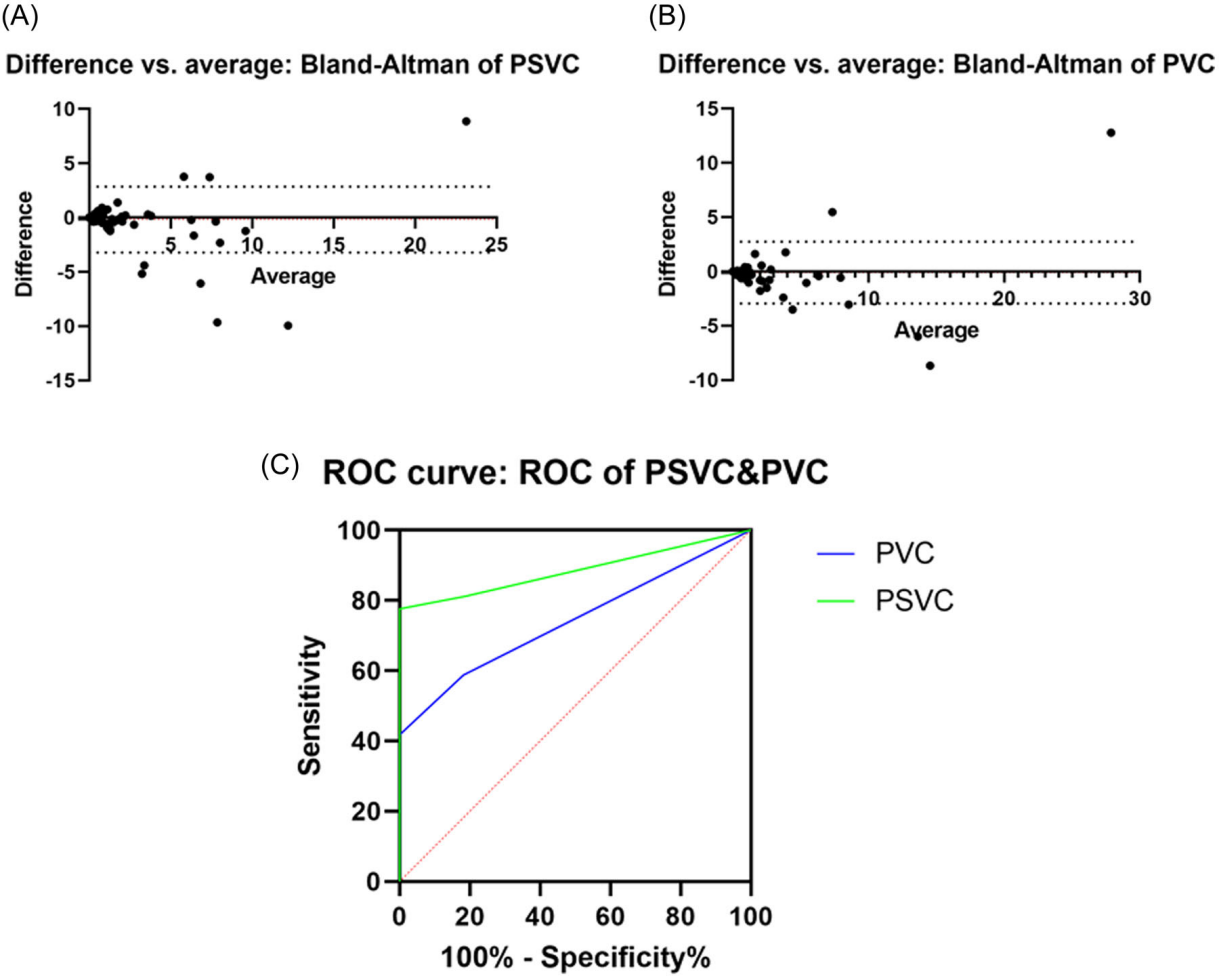
Illumination of diagnostic efficiency. (A) Results of Bland–Altman analysis in PSVC detection by 2 h‐Holter compared with 24 h‐Holter. (B) Results of Bland–Altman analysis in PSVC detection by 2 h‐Holter compared with 24 h‐Holter. (C) ROC curves for the sensitivity and specificity of the 2 h‐Holter in PSVC/PVC detection. The AUCs are listed in Table 4.

**Figure 2:**
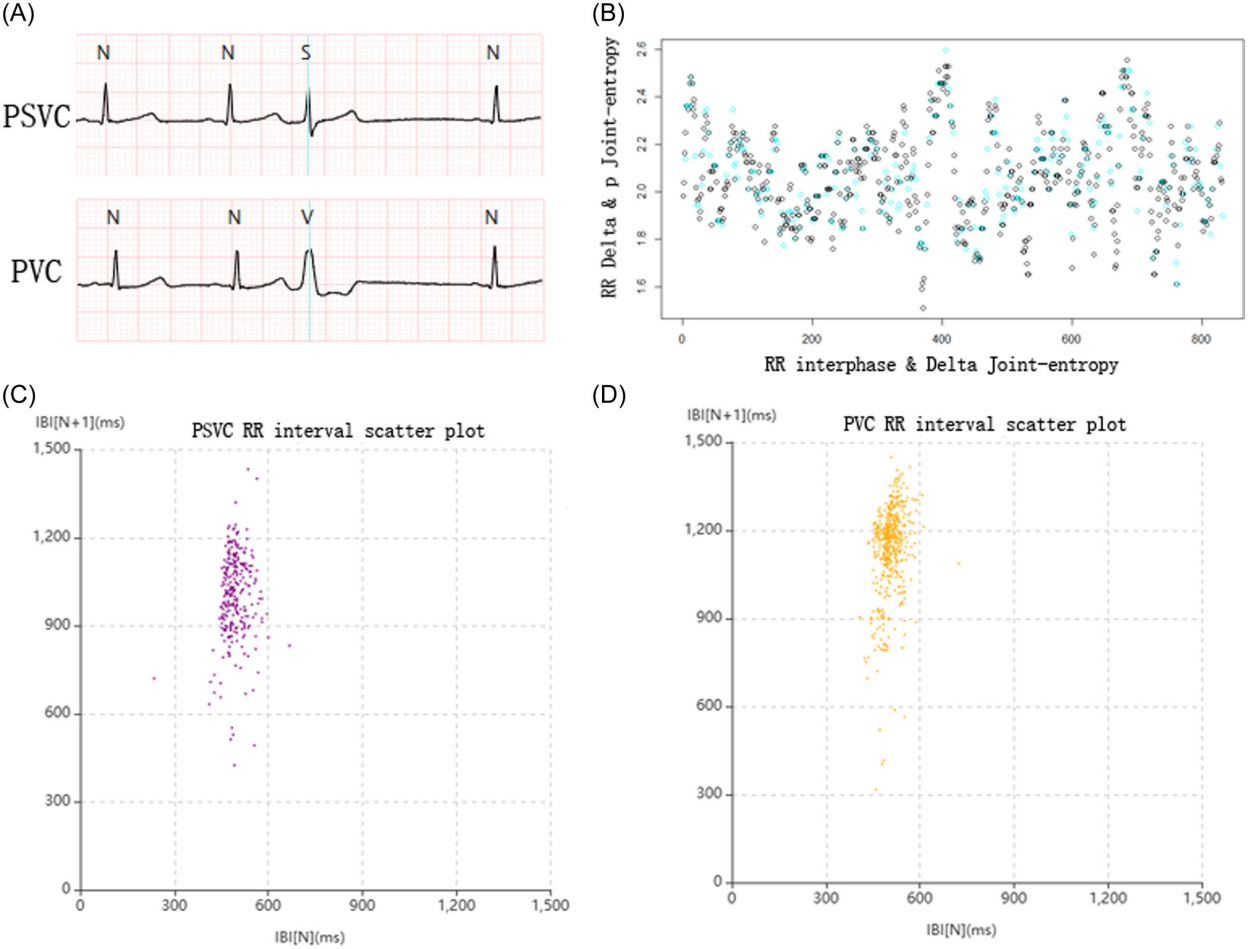
Exhibition of the AI‐based ECG signal capture and analysis. (A) Episodes of PSVC and PVC waves captured by the AI. (B) Simples of PVC detective training by 2 h‐Holter based AI platform (blue‐dots: PVC, black‐dots: normal beats). (C) PSVC scattergram based on inter‐beat interval (IBI) detection. (D) PVC scattergram based on inter‐beat interval (IBI) detection.

Following ECG, 24 h‐Holter and 2 h‐Holter wore during the same period. According to the reference standard of PSVC/PVC diagnosing, 24h‐Holter screened 115 patients without PSVC/PVC, 24 patients with PSVC, 23 patients with PVC, and eight patients with PSVC + PVC (Table 2). In the PSVC group, patients underwent 2 h‐Holter and were analyzed using the AI system, and the results were compared with 24 h‐Holter. PPV and NPV were 76.00% and 96.35%, respectively, and sensitivity and specificity were 79.17% and 95.65%, respectively. The PPV and NPV for the PVC group were 87.50% and98.55%, and the sensitivity and specificity were 91.30% and 97.84%, respectively (Table 3). ROC curves were used to further assess the diagnostic accuracy of the 2 h‐Holter in the PSVC/PVC Group (Figure 1). The AUCs are presented in Table 4. The results indicated an excellent AUCs for differentiating between the PSVC and PVC Groups. The AUCs values for PSVC and PVC were 0.885 and 0.741, respectively (*p* < .0001). Bland‐Altman analysis was used to compare the differences between 24 h‐Holter and 2 h‐Holter in the PSVC/PVC group, as shown in Figure 1. An image of a representative case with the application of a 2 h‐Holter is presented in Figure 2. AI‐captured episodes of PSVC/PVC ECG waves and RR interval scatter plots are also shown.

**Table 2:** Patient number and predict value of ECG/2 h‐Holter in PSVC and PVC groups.

Group	ECG (+)	ECG (−)	2 h‐Holter (+)	2 h‐Holter (−)
NC (115)	4	111	8	107
PSVC (24)	4	20	19	5
PVC (23)	6	17	21	2
PSVC + PVC (8)	5	3	8	0

*Note*: Holter 2 h (+): frequency ≥ 60/2 h; Holter 2 h (−): frequency < 60/2 h.

**Table 3:** Diagnostic value.

Modality	Sensitivity, % (95% CI)	Specificity, % (95% CI)	PPV (%)	NPV (%)
PSVC	79.17 (61.65–96.68)	95.65 (92.21–99.10)	76.00 (58.01–93.99)	96.35 (93.17–99.53)
PVC	91.30 (78.85–100.00)	97.84 (95.40–100.00)	87.50 (73.24–100.00)	98.55 (96.532–100.00)

Abbreviations: NPV, negative predict value; PPV, positive predict value.

**Table 4:** Parameters from the ROC curve analysis.

Modality	AUCs (95% CI)	*p*
PSVC	0.885 (0.846–0.924)	<.0001
PVC	0.741 (0.688–0.795)	<.0001

## 4 DISCUSSION

In a previous study, the value and limitations of ECG and Holter monitoring in the prognostic and diagnostic evaluation and therapeutic management of PSVC/PVC were the subject of abundant and often controversial literature.^7^ Our research also found that ECG was ambiguous in the PSVC/PVC diagnosis and burden analysis (data not shown). The 24 hours traditional Holter and 2 hours single‐lead Holter tests are both electrocardiogram (ECG) monitoring tests that are used to diagnose arrhythmias; however, few studies have investigated the differences in accuracy and diagnostic efficiency between these two methods.^21^ This study compared the aforementioned issues with a 24 h‐Holter and a 2 h‐Holter among patients requiring PSVC/PVC monitoring. The principal findings of the study are as follows: (i) During the simultaneous use of both methods, they yielded the same PSVC/PVC diagnostic efficiency in 24 or 2 hours intervals. (ii) The AI (CNN)‐based diagnostic support system provides new insights into the diagnostic application of wearable arrhythmias. (iii) Short‐time ambulatory monitoring (2 h‐Holter) captured PSVC/PVC episodes similar to the traditional ECG test, but were more accurate than the ECG test.

The major differences between our research and others are that (i) our research focused on the mid‐level diagnostic evaluation, the 2 hours single Holter device approved by the CFDA, which is different from the screened devices used in physical examination centers. (ii) The capture of PSVC/PVC episodes and burden analysis were based on the SWCAN Chinese ECG Database and MIT‐BIH Database. In this study, we have proven that a short wear period combined with a single‐lead Holter device would lead to advanced improvements in the following aspects: (i) making the acquisition process more convenient and less intrusive. (ii) leads to the wider adoption of arrhythmia screening in primary healthcare settings. (iii) improved diagnostic consistency compared with ECG.

Palpitation was the most common symptom in the cardiology department or other departments.^22^ The diagnosis and management of patients with palpitations, ECG test and 24 h Holter test are common. However, both ECG and 24 h‐Holter test are limited in medical care centers, and diagnostic limitations are obvious.^23^ Previous studies proved that the ECG test was only valuable for arrhythmia detection but ineffective for burden analysis.^24^ In our study, we found that the ECG test detected only 4/24 (16.7%), 6/23 (26.1%), and 5/8 (62.5%) patients with PSVC, PVC, and PSVC + PVC, respectively, compared to the 24 h‐Holter test. Therefore, management and diagnosis by ECG might have missed the diagnosis of PSVC/PVC. Indeed, the same efficiencies for 2 h‐Holter were 19/24 (79.2%), 21/23 (91.3%), and 8/8 (100%), respectively.

Recent guidelines recommend wearable devices for arrhythmia screen and management.^25^ The single‐lead Holter device was activated in recent years as the beginning of ambulatory monitoring. These accessible, feasible, and low‐cost devices have the potential for use by professionals. For instance, Zio‐Patches, apple‐watches, and other devices have exchanged detection methods for atrial fibrillation (AF), which depend on current signal capture and transmit technology.^10^, ^26^ The convenience of using the single‐lead Holter test was not only limited to the wearable style, but also to AI‐based analysis and wear‐time shortening. Compared with other wearable healthcare devices, the 2 h‐Holter device provided continuity detection and analysis. In contrast to the smartwatch and other optical pulse wave detection devices, the 2 h‐Holter device captured and analyzed the real‐time ECG signal, but not the optical analog signal. Different mechanisms would enhance the stability and accuracy of medical standards. In this study, a CNN‐based ECG signal analysis system was used to analyze PSVC/PVC waves and make a precise diagnosis. We aimed to reveal the real‐world efficiency of the AI system compared with that of cardiologists in PSVC/PVC diagnosis.

Several studies have investigated the use of a single‐lead Holter monitor for arrhythmia detection. Many studies have focused on the diagnosis of atrial fibrillation or tachyarrhythmias, particularly in handle devices or smartwatches. As well as multiple algorithms other than CNN were used for the ECG analysis. These include support vector machines, K‐nearest neighbors, and principal component analysis, and so on.^3^, ^6^, ^10^ Despite this, CNN has always shown high efficiency in ECG analysis, with advantages including feature extraction, translation invariance, scale and distortion tolerance, and other aspects. Indeed, PVSC/PVC‐related research has been insufficient. Therefore single‐lead Holter devices with AI diagnostic systems lack official recommendations. In addition, the short‐period record protocol, such as the 2 h‐Holter test has not been proven in previous studies. In our study, the combination of a single‐lead device and an AI analysis method was proven to improve the diagnostic efficiency of PSVC/PVC. Our data shown that the use of an AI‐based single‐lead Holter test could shorten the wear period and provide a new strategy for PSVC/PVC screening and diagnosis. Specially, in specific scenarios such as physical examination centers or primary health institutions, shortening the Holter acquisition time improves medical service efficiency. Which improved the patient comfort and compliance, accelerated diagnosis time, reduced cost efficiency and save medical data storage.

## 5 LIMITATIONS

The number of enrolled patients was small and this study focused only on inpatient/outpatient patients with palpitation. Therefore, diagnostic performance cannot be estimated in the general population. During the monitoring period, there were episodes of false negatives or false positives due to the single‐lead signal capture. The signal stability and accuracy can influence the analysis of AI systems. Additionally, the CNN algorithm was no longer the most advanced state‐of‐the‐art algorithm.

## 6 CONCLUSIONS

Compared with the standard traditional Holer test and ECG test, 2 hours single‐lead Holter test with AI analysis could provide accessible, feasible, and efficient detection rates for PSVC/PVC. The potential advantages of the 2 h‐Holter were shortened wearing period, improved convenience, and excellent consistency of diagnosis.

## AUTHOR CONTRIBUTIONS

**Xiangqing Kong, Ningtian Zhou, and Yiyang Zhan**: Designed, supervised the study and prepared the manuscript. **Yuansheng Fan, Jialing Wang**: Data collection and analysis, writing original draft. **Zhiyang Xu, Jialin Wang, Linfeng Yang, and Qiong Huang**: Data collection and analysis. **Qiong Huang, Ningtian Zhou, and Junhong Wang**: Writing—review and editing. **Xiangqing Kong, Yiyang Zhan**: Project administration and data analysis.

## CONFLICT OF INTEREST STATEMENT

The authors declare no conflict of interest.

## Data Availability

The data that support the findings of this study are openly available in AI in PSVC/PVC diagnosis at https://data.mendeley.com/datasets/y8dntyxhzc/1.
